# The improvised expert: Staging authority at an OECD Nuclear Energy Agency workshop in Fukushima

**DOI:** 10.1177/03063127241231822

**Published:** 2024-03-05

**Authors:** Makoto Takahashi

**Affiliations:** 1 Vrije Universiteit Amsterdam, Amsterdam, The Netherlands; 2 Cambridge University, Cambridge, UK

**Keywords:** improvisation, performance, public trust, expertise, Fukushima

## Abstract

In recent years, concerns about a crisis of expert authority have been expressed across the globe. Japan is no exception to this trend. Scandals surrounding the (mis)management of the 2011 Fukushima Daiichi nuclear power plant disaster severely damaged public confidence in state institutions, posing an additional challenge for those engaged in radiological protection. This article examines how claims to expert authority are made in these conditions of low public trust. To this end, I offer an ethnographic account of the OECD Nuclear Energy Agency’s (NEA) *Workshop on Post-Accident Food Safety Science*—an event staged at the request of the Japanese Cabinet Office with the aim of inspiring confidence in Fukushima produce. I analyse the practices through which the organizers craft a credible public persona using the idiom of dramaturgical improvisation; drawing attention to the ‘performed resourcefulness’ with which they adapted extant institutional scripts in response to a discerned crisis of public reason. Concretely, improvisation invites us to consider how and why nuclear policy actors have sought to demarcate two variants of the deficit model: the (psychological) discourse of ‘radiophobia’ and the (economic) discourse of ‘reputational damage’. Where prior scholarship has identified the continuities between the two discourses, an attention to this boundary work reveals the dramaturgical advantages of ‘reputational damage’ over ‘radiophobia’ in contesting critics’ claims to the mantle of victimhood, securing international support, and producing the expert’s body as a site of evidence.

No one was meant to drink the water that passed Sonoda Yasuhiro’s lips on 31 October 2011. This puddle-water had been taken from the floors of the Fukushima Daiichi nuclear power plant and decontaminated for uses such as cleaning ash from trees nearby to prevent forest fires. How then, had a member of the Japanese House of Representatives come to decant it into a drinking glass? Our story begins days earlier, at another press conference. Addressing the assembled reporters in his capacity as a spokesperson for the Cabinet Office, Sonoda had struggled to dispel concerns regarding the decontamination process. Having insisted that the liquid was ‘safe enough to put in your mouth’, Sonoda was swiftly challenged to put it in *his* mouth, leaving him little choice but to imbibe. ‘If that’s the best way to prove that the water is safe,’ he replied, ‘then I would gladly drink it right here, in front of you, any time.’ Raising the glass with a concerned gaze and trembling hand, the politician paused—aware, perhaps, that this would become an ‘iconic image’, shared by news outlets across the globe^1^ as a pivotal episode in the ‘battle to convince a sceptical world that the Fukushima crisis is under control’ (McNeill, 2011). With hopes of inspiring confidence in the Cabinet Office’s assurances, he threw back his head and drank.

Sonoda insisted that he did not want his actions ‘to be seen as some kind of performance’ and noted that ‘just drinking [the water] doesn’t in itself mean safety is confirmed’ (in McCurry, 2011). The ‘best way’ to do that is ‘to present data to the public’, he insisted. Yet, his decision to stage this event, under the strobe light cast by the cameras of the press, speaks to the dramaturgy of epistemic controversies. As scholarship in the field of Science and Technology Studies (STS) has emphasized, authority is not a right, automatically granted to those who demand or deserve it. To have their claims accepted as authoritative, those who engage in public debate do more than just ‘present [their] data’; they carefully craft personas that convey virtues such as credibility, competence, and integrity (Hilgartner, 2000). Many of the practices through which they do so are prosaic, but Sonoda’s actions reminds us of how *emotive* credibility contests can be. Sonoda aimed to inspire both trust and a ‘sense of safety’ (*anshin*). He was undone by the tremor in his hand.

This article examines how claims to expert authority are improvised in conditions of low public trust. To this end, I offer an ethnographic account of the OECD-NEA’s *Workshop on Post-Accident Food Safety Science*, held in Fukushima from 8 to 10 November 2016, at the request of the Japanese Cabinet Office. I focus on an object of analysis that is, by nature and necessity, a public affair: the *persona* of the NEA workshop. Following Hilgartner (2000), I seek to draw attention to the importance of crafting a credible persona to the exercise of authority; working from the premise that our willingness to accept advice is influenced by our perception of its source. The purpose of this ethnography is to document the micro-social processes through which this persona is constructed and maintained. But where Hilgartner uses the rubric of *scripts* to foreground the consistency with which trusted actors enact established personas, I mobilize the idiom of *improvisation* (Greenblatt, 1980; Jeffrey, 2013), to draw attention to the ways in which the Cabinet Office and OECD-NEA have responded to a perceived crisis of trust in their authority. In so doing, I emphasize the ‘performed resourcefulness’ (Jeffrey, 2013) with which policy professionals imbue their actions with credibility, not simply by enacting standardized scripts, but by crafting personas that meet the demands of a specific political moment. Concretely, I show how adopting an economic register has allowed the Japanese state to mobilize international supporters, with their associations of dispassion and objectivity, and that the focus on economic objects is more amenable to what Ezrahi (1990) calls an ‘attestive visual culture’ than a focus on psychological subjects. Anxiety and depression remain private experiences, therapy taking place behind closed doors. However, food stuffs can be tested and consumed publicly, allowing food safety science to be ‘produced and confirmed through public witnessing’ (Ezrahi, 1990).

In the wake of the 2011 Fukushima Daiichi nuclear disaster, Japanese politics has been characterized by widespread concern about radiation exposure and scepticism about the state’s assurances of safety. This atmosphere of distrust has proven an obstacle to the government’s ongoing efforts to reconstruct the affected territories (*fukkō*). As the Tohōku region is famed for its agricultural produce, restoring confidence in food from Fukushima has been deemed essential to reconstruction. To this end, the state responded to the perceived laxity of the food safety standards introduced in March 2011 by introducing significantly stricter thresholds, agreed through the Food Safety Commission in April 2012. These newer standards are considered conservative by international standards, but widespread aversion to Fukushima produce persists both domestically and internationally. As Sternsdorff-Cisterna (2015, p. 456) reminds us, ‘food safety is both a question of science and of affective networks of trust. Food must be safe (*anzen*) and feel safe (*anshin*)’ (see, also Fassert & Hasegawa, 2019). A confused emergency response and evidence of a culture of cosy collusion between government, regulators, academics, journalists, and industry made it difficult for political authorities and experts alike to inspire this ‘feeling of safety’ in Japan (Kingston, 2012; see also: NAIIC, 2012). It is with hopes of fostering trust in the state and combatting the ‘harmful rumours’ causing ‘reputational damage’ to Fukushima’s produce that the Cabinet Office commission the OECD-NEA workshop.

Improvisation allows us to see how and why—in seeking to achieve this *feeling* of safety—nuclear policy actors have sought to demarcate two variants of the deficit model: the (psychological) discourse of ‘radiophobia’ and the (economic) discourse of ‘reputational damage’. Prior scholarship has identified the continuities between these two discourses, which posit the (irrational) fear of radiation as a threat to public health and the economy of the affected territories, respectively. Both discourses task the state with achieving the ‘management of emotions’, either by lowering Fukushima residents’ stress about exposure or overcoming consumers’ fear of Fukushima produce. Nonetheless, the two discourses remain distinct in the eyes of the radiation protection community. Like many reports and workshops produced or commissioned by the Japanese state, the NEA workshop focuses on tackling ‘reputational damage’ while carefully avoiding the language of radiophobia. Attention to this boundary work reveals the *dramaturgical advantages* of the economic frame over its psychological counterpart in managing the Fukushima Daiichi disaster. It is not just that framing the fear of radiation as pathological (i.e. a phobia) can alienate potential allies in the international community; it is also that, despite their commonalities, radiophobia and reputational damage construct different subjects, objects, and audiences, and (consequently) imply notably different affective politics—transitioning from one discourse to the other moves the mantle of victimhood and the locus of sympathy. Radiophobia frames its audience as hysterical *victims* of their own irrationality; reputational damage frames its audience as *victimising* the farmers of Fukushima through their irrational consumption habits. One should *feel for* the radiophobe while those causing ‘reputational damage’ should *feel for* the farmers of Fukushima.

## Dramaturgical performance and improvisation

The analysis of personas can be traced back at least as far as Goffman (1959/1990); see Turner, 1982). For Goffman, life is not drama, as such. He emphasizes life’s similitude to theatre, not to make assertions regarding the nature of being, but to invite us to examine how actors present themselves in their daily interactions, much as a theatre critic might dissect the techniques that thespians use to embody particular characters (Branaman, 2013).

Contemporary STS’s interest in Goffman (1959/1990) can be credited to Hilgartner’s (2000) *Science on Stage*. Applying the dramaturgical metaphor, not to the activities of individual actors, but to the personas of scientific advisory bodies, Hilgartner (2000) sought to identify core elements of the United States National Academy of Science’s (NAS) ‘institutional script’ for staging authoritative scientific reports. These elements include: (a) the use of persuasive rhetoric (pp. 5-48); (b) the establishment of a vouching network (pp. 50); (c) the forging of a single voice (pp. 51-52); and (d) the clear delineation of a visible ‘frontstage’, where conclusions are presented, from a secluded ‘backstage’, where much of the preparation and deliberation takes place (pp. 52-85). Although, Hilgartner’s singular focus on NAS reports has led critics to question the representativeness of his conclusions, especially regarding the separation of ‘front’ and ‘back’ regions (Bijker, 2002; Campbell, 2004), each of the practices identified by Hilgartner can be found (in some fashion) in the OECD-NEA’s performance of authority at its workshops.

### The Fukushima disaster as a crisis of public trust

From the beginning, the Fukushima Daiichi disaster has been understood to be both a nuclear crisis and a crisis of trust in the Japanese state (Kumaki, 2024). A few days into the crisis, a Reuters headline announced, ‘Japan government losing public trust as nuclear crisis worsens’ (Sieg, 2011). *The Economist* (2011) followed a few months later with the headline, ‘A question of trust: Japan’s nuclear crisis is eroding deference to authority’. By the following year, public relations firms like Edelmann (2012, p.6) were announcing that the disaster had exposed ‘the fragility of trust’ in Japanese institutions, pointing to dramatic declines in how many surveyed citizens expressed trust in the government, business, media, and NGOs or regarded government officials or academics as credible spokespeople (see also Genron, 2019). By the following year, this distrust was manifested in the largest protests Japan had seen in half a century (Oguma, 2017)—and civil servants would walk home past weekly demonstrations outside the Prime Minister’s Residence for years to come (Takahashi, 2020, pp. 1–2). A decade later, many retrospectives penned for the decennial emphasized that Japan is still ‘repairing damage and public trust 10 years after Japan’s triple disaster’ (Kuhn, 2021). Where NAS committees act in the knowledge that the failed staging of a report can *become* an issue of ‘whether the Academy is a trustworthy advisor to government’, the Japanese state has staged its claims to credibility aware that their status as ‘a responsible guardian of the cultural authority of science’ is *already* in question (Hilgartner, 2000, p. 113).

Challenges to the Japanese state’s credibility have overwhelmingly been made in the language of corruption, cronyism, and revolving doors. The retirement of elite bureaucrats into industry is romantically dubbed ‘descent from heaven’ (*amakudari*) and scholars supportive of government positions swiftly find themselves labelled ‘academic flunkies’^2^ (*goyō gakusha*), in reference to those intellectuals who sold their services to the Tokugawa shogunate. Yet the term that defines the post-Fukushima period is the ‘nuclear village’ (*genshiryoku mura*): an imagined collective of politicians, bureaucrats, utility officials, academics, and journalists, bound by a shared interest in promoting nuclear power and a lattice of *quid pro quos* (Kingston, 2012). Tellingly, each of these terms strikes not at the competence of state officials, but their rectitude. In a political culture that emphasizes probity and civic duty, these accusations of self-interest have cut deep. The issue—as one independent inquiry put it—is that ‘bureaucrats … put … organizational interests ahead of their paramount duty to protect public safety’ (NAIIC, 2012, p. 9). It is the suspicion that Japan’s food safety standards are products of the same self-serving elite—and designed to downplay the disaster’s effects and minimize compensation payments—that has made it so difficult for the state to inspire the ‘feeling of safety’.

### Improvisation

In analysing how actors establish claims to authority in the absence of trust, scholars in literary theory (Greenblatt, 1980) and political geography (Jeffrey, 2013) have independently reached for the vocabulary of *improvisation*. Defined by Greenblatt (1980) as ‘the ability both to capitalize on the unforeseen and to transform given materials into one’s own scenario’ (p. 227), improvisation is an idiom that frames credibility contests as exercises in power, rather than virtue. Its most important feature is not spontaneity, but a conscious self-fashioning aimed at inspiring trust. In this, it differs from the routinized roles that actors play in deference to social or professional norms. To be said to be improvising, one must act with the intention of ‘inserting oneself into the consciousness of another’ (p. 227). Colonial Europeans efforts to ingratiate themselves with indigenous populations are Greenblatt’s most famous example (pp. 224-229). Thomas More’s efforts to turn the public against Protestant reformers is another (pp. 229-232). Here, I treat contemporary efforts to combat a (perceived) crisis of trust as an analogous case, in which credibility is once again ‘a major occupation, the object of cultivation and fear’ (p. 227).

Improvisation does not displace other modes of dramaturgy; it sits alongside them. In the NEA workshop, one can see both a workshop script, common to all OECD workshops, and gestures crafted for the post-3.11 context. Actors may not recognize the former as theatrical, having normalized them as part of how such events work, but the latter are consciously performed with a sceptical audience in mind. In these gestures, we see a ‘willingness to play a role, to transform oneself, if only for a brief time’ in the name of establishing trust (Greenblatt, 1980, p. 228). By including both staged and improvised gestures in this analysis, I move the focus away from actors’ efforts to stick-to-the-script, (which is central in Hilgartner’s (2000) work), and foreground the ‘performed resourcefulness’ with which actors add to and embellish scripts instead (Jeffrey, 2013). In so doing, I emphasize the ‘emotional’ (Hochschild, 1983) and ‘affective labour’ (Hardt & Negri, 2000) expert bodies do to read their audience and adapt how they present themselves. Weinberg (2020) calls this ‘false-face acting’; Greenblatt dubs it ‘self-fashioning’. Good improvisation necessarily demonstrates a grasp of dominant *styles of public reason*—for only if actors are attuned to ‘tacit knowledge-ways through which they [societies] assess the rationality and robustness of claims’ (Jasanoff, 2005, p. 255) can they hope to persuade. As I demonstrate, experts in radiological protection are keenly aware of this need for contextual sensitivity—in the EC et al.’s (1996) words, the successful ‘management of emotions’ requires that experts read the ‘emotional context’ of the post-accident situation and act accordingly (p. 96).

## Background and methods

### The OECD Nuclear Energy Agency

The *Workshop on Post-Accident Food Safety Science* was organized by the OECD-NEA at the request of the Japanese Cabinet Office. The NEA is a fitting organization to study, both because it is widely recognized as an authoritative source of technical policy advice, and because it is an institution of historical significance, which has received little academic attention. Founded in 1958 as the European Nuclear Energy Agency (ENEA), it played an important role in laying the foundations for international cooperation in the nuclear sector during the mid-20th century. Changing its name in 1972, it continues to act as a nexus of knowledge in the global circulation of nuclear expertise. Among the NEA’s 33 member states are 21 of the world’s 31 nuclear nations, who collectively account for 354 of the world’s 451 operational nuclear power reactors and 85% of the world’s nuclear energy supply.^3^ As these member states act as both customers and donors to the NEA—commissioning its research and staffing its seven technical committees—its activities can be seen as a barometer for the interests and ideas circulating throughout the regulatory community. For this reason, the NEA has a place in any full account of the global ecology of nuclear institutions, alongside organizations such as the International Atomic Energy Agency (IAEA) and the International Commission on Radiological Protection (ICRP).

This article focuses on the OECD-NEA not as a historical object, but as an authoritative source of technical policy advice. The OECD plays a significant role in harmonizing science, technology, and trade policy among its member states through means of ‘soft law’: non-binding recommendations, metrics, and frameworks (Frahm et al., 2022; Laurent, 2016). Its work is widely recognized as a credible basis for policy, not just by its 37 member states, but by other international organizations—such as the IAEA, World Trade Organization (WTO), UN Food and Agriculture Organization (FAO), and European Commission—giving it a credible claim to a global policy reach (Mahon & McBride, 2009; Woodward, 2009). The WTO, for example, notes that research conducted by the OECD Secretariat is ‘regularly used by WTO Secretarial Staff to perform its duties’ and is often ‘instrumental in preparing future negotiations at the WTO’ (WTO, 2023). The issue of post-Fukushima Daiichi food safety is no exception and assessments provided by the NEA are among the sources cited by the WTO Secretariat in preparation for dispute resolution between Japan and Korea. Lacking the formal powers of a regulatory body, the NEA exerts its influence through the force of its reputation. Hence, its *Mission Statement* focuses on ‘provid[ing] *authoritative* assessments … on key issues’ as the means by which to effect ‘government decisions on nuclear energy policy’ (OECD-NEA, 2004, p. 2, emphasis added). But, as political pragmatists from the time of Machiavelli (1883/1970) onwards have warned, reputation is a delicate thing, and an institution must constantly ‘give signal proofs of [its] worth, whether by words or by deeds’, so as to ‘pass into proverb’ (p. 523). As such, the NEA must strive to perform the virtues of authority and credibility, on which its reputation rests, at any given public-facing event—allowing the ethnographer to document its practices of impression management.

### Workshop on Post-Accident Food Safety Science

Commissioned by the Japanese Cabinet office, the *Workshop on Post-Accident Food Safety Science* was held on 8 to 10 November 2016, at CORASSE Fukushima: a complex located by the West Exit of Fukushima Station, which stocks a variety of locally produced goods in addition to serving as a conference venue. The event was funded by the Japanese Cabinet Office and attended by no fewer than five Japanese politicians: former Vice-Minister of the Cabinet Office and for the Environment, Mamoru Fukuyama; his immediate successor, Inoue Shinji; his incumbent succesor, Tadahiko Itoh; Deputy Governor of Fukushima Prefecture, Toshiyuki Hata; and Masako Mori, a member of the House of Councillors born in Fukushima.

The Japanese Cabinet Office asked the NEA (2016)^4^ to review ‘the state-of-the-art scientific aspects of post-accident food safety’ (Workshop Objective 1) and ‘the status of the remaining challenges’ in Japan (Objective 2) with the aim of eliminating *‘reputational damage’*: influentially defined by workshop participant and Session Vice-Chair, Sekiya (2003) as ‘economic damage due to … disrupted consumption … as a result of people viewing foods, products, and localities once deemed safe as being dangerous because of widespread media coverage of … a disaster’ (in Monma et al., 2015, p. 222). Each of the Japanese Cabinet ministers in attendance expressed their ‘hope that this workshop will lead to [the] eliminat[ion of] reputational damage in food and that it will be a big step toward the future of Fukushima, full of possibilities’ (Itoh, 2016; see also Fukuyama, 2016; Inoue, 2016). The OECD NEA echoed this aspiration. Though its representatives stressed that the agency does not seek to ‘command on Japanese regulatory standards of food, since they are directly national choices’ (Iracane, 2016), they nonetheless hoped that the workshop would help inspire consumer confidence:

[W]hilst the safety [of food produced in Fukushima] is *guaranteed* by producer and the government, it is clear that there remain local, national, and international barriers to the confidence that existed in all Japanese food, prior to this accident. To shed a scientific light on the situation, the Japanese government asked the NEA to organize a workshop … on the state of the art in food safety science … *I hope that this will help to improve confidence in governmental, distributor, and farmer’s efforts to guarantee the highest quality food product from Fukushima prefecture.* (Iracane, 2016, emphasis added)

The core assumption shared by the Japanese state and NEA is that food from Fukushima is safe. In April 2012, Japan significantly tightened its food safety standards. The new Food Safety Commission standards superseded the provisional measures adopted in March 2011^5^ and sought to lower exposure through ingestion from 5 mSv/year to just 1 mSv/year.^6^ Japan has said that its standards are ‘among the strictest in the world’—and although direct comparison to other frameworks is complicated, these thresholds are considered conservative by international standards. Acting under the assumption that actors avoid Fukushima produce because they do not know how strict Japanese food safety standards are, the cabinet office commissioned the workshop to establish ‘the facts’ about Fukushima produce, thereby ‘facilitating the accurate perception’ of ‘the hard work undertaken by [Japanese] farmers, distributors, and municipalities’ to ensure that Fukushima products are ‘very safe’ (Inoue, 2016).

### Data collection

My analysis of how the OECD-NEA crafts a credible persona is grounded in participant observation of the workshop itself, conducted as part of a four-year project on the politics of civilian radiation exposure which entailed 18 months of residential fieldwork and interviews with 47 politicians, scientific advisors, bureaucrats, NPO representatives, protestors, and public figures engaged in this epistemic contest. As the workshop is an ephemeral space, lasting only three days, the duration of my observation was necessarily short. However, the very fact that direct observation was possible should be considered a boon. Access to the workings of scientific advisory bodies, such as commissions or report committees, can be difficult to secure. Consequently, many scholars have restricted their analyses to published materials (Hilgartner, 2000) or archival documents (Owens, 2012), even as they acknowledge that ‘ethnographic observation is without a doubt the method of choice’ (Hilgartner, 2004, p. 446). The volume of material produced by the workshop should also not be underestimated. The formal sessions alone account for an estimated 106,000 words.^7^ Supplementing detailed ethnographic field notes and video recordings of the proceedings with interviews and textual materials (e.g. workshop programme, OECD-NEA website, etc.) produced a robust empirical corpus on which to draw.

## The Workshop’s architecture of authority

### The rhetoric of reputational damage

As Kimura (2016) argues in *Radiation Brain Moms*, ‘reputational damage’ is often deployed as a term of abuse, used to police expressions of concern about food safety. A compound of ‘harmful rumour’ (*fūhyō*)^8^ and ‘damage’ (*higai*), reputational damage (*fūhyō-higai*) is a Japanese legal term that emerged in the 1980s (Sekiya, 2003, 2011). It was coined to fill a gap in nuclear liability law, offering a mechanism for producers (and tourist destinations) to be compensated when the association with a nuclear event caused consumer aversion to a product, without causing substantive damage. To say that a product is suffering reputational damage is necessarily to claim that it is safe in the eyes of the law and that consumers’ fears are groundless: nothing more than ‘harmful rumours’.

Reputational damage entered common parlance in the wake of the Fukushima disaster, its usage closely paralleling (and often overlapping) with the discourse of ‘radiophobia’: a term that emerged in the USSR following the Chernobyl disaster and used to denote a pathological fear of radiation. The unwieldy Japanese transliteration and translation of ‘radiophobia’ (*hōshanō kyōfu shō*), deployed by scientific advisors to Fukushima Prefecture in their public addresses (e.g. Yamashita, 2011), has seen less popular use than a vernacular equivalent, ‘radiation brain’ (*hōshanō*^9^)—a homophonous pun on ‘radiation’ (*hōshanō*^10^). Nonetheless, both radiophobia and reputational damage have been prominently mobilized by representatives of the Japanese state, and magazine headlines like ‘‘Walking reputational damage, [Councillor] Yamamoto Taro’s ‘radiation brain’’ speak to both the popular uptake of these discourses and their frequent imbrication (Sakai 2021).

Despite their common features and overlapping usage, a close reading of the NEA workshop invites us to consider how the two discourses remain distinct in the minds of the expert community and how the discourse of reputational damage may offer dramaturgical advantages that radiophobia does not. It is notable that in convening the NEA workshop, the organizers carefully avoided any mention of ‘radiophobia’—mirroring a broader aversion to the term that one can observe in Ministry of Agriculture, Forestry, and Fisheries (MAFF) and Ministry of Environment (MoE) documents. Radiophobia is a medicalising discourse, which posits that the most profound danger to public health is not radiation but fear. Aversion to low doses of radiation is read as both a direct detriment to mental health and as informing choices which indirectly affect actors’ mental and physical wellbeing. Concretely, the fear of radiation is understood as a cause of anxiety and depression, as well as a root of decisions not to return to Fukushima. For many, this has meant living in temporary housing for extended periods. In Japan, the use of this term is most commonly associated with Fukushima Medical University and the staff seconded from Nagasaki and Hiroshima Universities (e.g. Yamashita, 2011).

**Figure 1.**
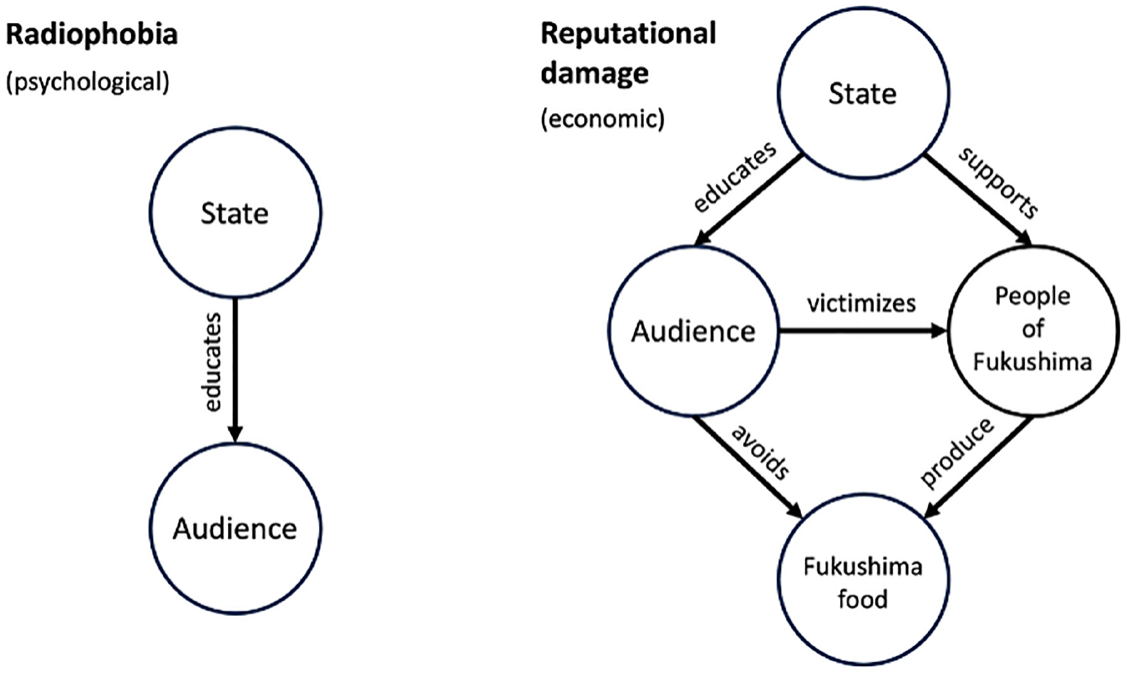
Rhetorical structures of radiophobia and reputational damage.

By contrast, reputational damage is an economic and legal frame. Its principal object is the goods and services of Fukushima prefecture. Its subjects are not the people of the prefecture (many of whom preferentially purchase local produce) but those who live outside it. This expands the geography of the audience, from strictly domestic (and predominantly regional) to both domestic and international. Moreover, it changes the character of the audience and its relationship to the mantle of victimhood. The rubric of radiophobia constructs two subjects: the science communicator and her audience, the ‘hysterical’ victims of misinformation. The former corrects deficits in the latter’s understanding of science to promote their wellbeing. By contrast, the rhetoric of reputational damage implies three subjects (science communicator, audience, and food producers in Fukushima) whose relations are mediated by an object (Fukushima produce). In this frame, the audience of consumers and gatekeepers (e.g. foreign states, food distributors) are no longer victims. Instead, they cause others to suffer. The locus of sympathy is thus shifted away from the imagined audience to the farmers of Fukushima (Figure 1).

The moral orders implied by radiophobia and reputational damage powerfully shape the terms on which actors can engage those who challenge their authority (Table 1). Crucially, the discourse of radiophobia centres the emotional state of those who express concern about radiation exposure. To claim that such critics are ‘radiophobic’ assuredly brings their ability to reason into question. Yet it also grants them the status of victimhood. ‘People … *suffer* … from radiophobia’ (Yamashita, 2011, emphasis added). The implied moral imperative is to alleviate this plight; ‘to lower the anxiety (and) give them emotional support’ (Yamashita, 2011). The science communicator is tacitly obligated to perform empathy for her (psychologically distressed) critics. One should *feel for* the radiophobe. No such obligation is implied by the frame of reputational damage. To the contrary, the burden of empathy is displaced from the science communicator onto her audience. In Minister Itoh’s (2016) account of the NEA workshop, the problem is not only that consumers are ignorant of the facts but also that they are not aware of the harm that they cause. They do not see how ‘tormented’ the fishers and farmers of Fukushima are by harmful rumours (Yamashita, 2011). To ‘know’ that Fukushima produce is safe thus becomes a form of civic duty. The good citizen or organization *feels for* those directly affected by the disaster (*tōjisha*) and recognizes their complicity in aiding—or hindering—Fukushima’s reconstruction. As the OECD reflected three years later, the 2016 ‘symposium emphasised that it is now a crucial matter of *responsibility* and *solidarity*… to buy, sell and promote products ‘from Fukushima’’ (OECD-NEA, 2022, p. 5, emphasis added). Where scientists encounter those who trenchantly refuse this ‘duty’, they can dismiss them to focus on those they are more likely to persuade. One research informant from a citizen science group recounted how she had sought to question the safety of food from Fukushima following a town-hall meeting on reputational damage. The presenter flatly responded that they were ‘not trying to convince people like her’ and were aiming to reach the other ‘80% of people’.

**Table 1.** Radiophobia and reputational damage—a comparative view.

	Radiophobia	Reputational damage
Epistemology	Psychology, public health	Economic, legal
Coinage	USSR, 1980s	Japan, 1980s
Associated institutions in Japan	Fukushima Medical University (FMU); Fukushima Medical Health Survey (FMHS)	Japanese courts; Tokyo University; Fukushima University
Prominent proponents	L.A. Ilyin & O.A. Pavlovsky; Shunichi Yamashita	Naoya Sekiya; Ryota Koyama
Victims	Concerned laypeople	Food producers in Fukushima, tourist destinations
Audience	(Domestic) victims	Consumers (outside Fukushima), food distributors, foreign states

#### Vouching

The persona of the workshop is also established through a second mechanism that Shapin (1988, pp. 388-404) and Hilgartner (2000) refer to as *vouching*. Following Hilgartner, one can argue that by appointing Naoya Sekiya as an Associate Professor, Tokyo University vouches for his character and competence (p. 50). And by allowing his name to appear on the workshop materials as a Session Vice-Chair, Sekiya implicitly vouches for the workshop and the validity of its conclusions (p. 50). ‘To be sure, no representative’ of Tokyo University reviewed the final statement of its conclusions ‘on the university’s behalf’, but the workshop ‘created a chain of associations through which the reputational resources’ of Tokyo University can be mobilized (p. 50). Multiple vouching-practices can be seen at play in the NEA workshop. The circulation of the agenda alone—which lists the name of each chair, presenter, and rapporteur with a corresponding institution (e.g. ‘Igor Gusev, IAEA’)—links the event to no less than 28 organizations, including: two Japanese ministries; two Japanese state agencies; three eminent international organizations; seven foreign state agencies; and four academic institutions (see Table 2). This network of associations is systematically reinforced during the floor discussion, by the compulsory practice of stating one’s name and affiliation prior to offering a point. While the practice of providing a short, professional biography of each chair and presenter establishes an even more expansive vouching-network: linking the participant to their past, as well as their present, places of employment.

**Table 2.** Organizations listed on the Workshop Agenda.

Institution type	Institution
*International organizations*	• IAEA • ICRP • OECD-NEA
*Japanese Ministries*	• Ministry of Agriculture, Forests and Fisheries (MAFF) • MHLW
*Japanese State Agencies*	• Japanese Atomic Energy Agency (JAEA) • Nuclear Regulation Authority (NRA)
*Local Governments*	• Fukushima Prefecture
*Prefectural Organizations*	• Fukushima Agricultural Technology Centre • JA Fukushima Mirai • Fukushima Prefectural Federation of Fisheries Co-operative Associations
*Local associations and co-operatives*	• Fukushima Beef Promotion Association • Co-op Fukushima
*Foreign State Agencies*	• Centre for Environmental Fisheries and Aquaculture Science (CEFAS), UK • Environmental Protection Agency (EPA), US • Institute of Radiological Protection and Nuclear Safety (*Institut de radioprotection et de sûreté nucléaire*) (IRSN), France • Norwegian Radiation Protection Agency (NRPA), Norway • Public Health England (PHE), UK • Research Institute of Radiology (RIR), Belarus • SCK-CEN, Belgium
*Universities*	• Fukushima University • Oita University of Nursing and Health • Tokyo University • University of Cumbria

Source: OECD-NEA Workshop Agenda.

By avoiding the terminology of ‘radiophobia’, the workshop is able to mobilize an international (vouching) network. Despite its prominent use by Japanese science advisors, radiophobia is a controversial term within the international community. (A senior member of one international organization confessed that they had been ‘surprised’ to see the Japanese state revive the term.) Many contemporary members of the NEA—and other international organizations in the nuclear domain (e.g. ICRP and IAEA)—contributed to the *International Chernobyl Project* (1991) and *One Decade After Chernobyl* (1996) conference, sponsored by the EC, IAEA, and WHO and chaired by then-German Minister for the Environment, Nature Conservation and Nuclear Safety, Dr. Angela Merkel (see Takahashi, 2020, pp. 134-140). The consensus position of both projects was to condemn radiophobia on moral and practical grounds. The *International Chernobyl Project* (1991), for example, reported that ‘distrust of both scientific and political authorities … was not helped by officials who began attributing public fears to ‘radiophobia’ or undue concern about radiation and its health effects’ (p. 52). In the view of the authors, concern about exposure to radiation was reasonable, even if it was (statistically) disproportionate. ‘If the stressor is a real threat it is *dishonest* to pretend otherwise or to imply that an anxious response is in some way abnormal’ (p. 348, emphasis added). ‘False labelling such as … “radiophobia” is counterproductive’, one prominent contributor to the *One Decade After Chernobyl* conference agreed (in EC et al., 1996, p. 217). To rebuild confidence, risk must be communicated to affected peoples ‘without downplaying the damage that has been done and without implying that their reactions are pathological (e.g. radiophobic)’. Given these public denunciations of the term, such actors would be unlikely to lend their reputations to a workshop framed around combating radiophobia. Avoiding the register of psychology, in favour of a more palatable economic discourse, is therefore key to the workshop’s ability to both reach international audiences and mobilize their support (Table 2).

### Forging a single voice

A third move through which the workshop seeks to assert the credibility of its conclusions, is to forge a ‘single voice’, which focuses the social capital of the organization’s vouching-network onto its findings. The mechanisms through which this is achieved differ markedly from the production of scientific. Unlike the textual report, workshops are, by nature, poly-vocal affairs. Attendees alternate in contributing as individuals, not as a collective. To overcome this hurdle, the workshop makes two related moves. One, it tacitly adopts a model of passive consent, broadly consistent with the *Rules of Procedure*, which govern the OECD’s written documents (OECD, 2013, § 6). The presentations that comprise the bulk of the workshop are interspersed with floor discussions. These are scheduled for the end of each session and last between 10 and 30 minutes. As these occasions provide an opportunity to object to any point made by another participant—albeit an opportunity limited by time and social convention (more later)—the onus is placed on participants to make any reservations they have known. To say nothing, as the proverb states, is to consent. Hence, the claim of a single participant can, through audience inaction, come to be a workshop conclusion—able to draw on the combined social capital of the participating individuals and institutions. Secondly, the workshop appoints certain individuals with the task of actively constructing a consensus, namely the Session Chairs and Rapporteurs. Chairs are vested with the authority to shape the floor discussions, steering the debate by dictating who is given an opportunity to speak and when. They comment on presentations and themes, framing the debate as a whole. They can also decide which conversations fall beyond the scope of the workshop aims and when discussion can be brought to a close. In short, they are vested with the privileges of an editor of the debate. More obviously, the rapporteurs quite literally speak for the whole—providing a single authoritative interpretation of the workshop discussions.

### Stage management

The workshop can only speak with one voice if the tensions, contradictions, and disagreements between its contributors can be hidden. Contributors must therefore co-operate in maintaining a division between the ‘frontstage’—in which the workshop presents its arguments and conclusions to its audience—and the ‘backstage’—in which preparatory work is conducted and disagreements resolved. Central to Hilgartner’s analysis of how the NAS’ persona is constructed, is an analysis of the techniques through which this scientific advisory body ‘backstages’ disagreement. Campbell (2004, p. 439) argues that ‘the secretive processes of the NAS … may limit the utility of [Hilgartner’s] analysis for other, more public bodies and even other NAS committees’. This is a legitimate concern, given that the NAS is unique among its peers in its exemption from Freedom of Information requests, and is unusual in its dedication to opacity. Few other scientific bodies systematically ask contributors to sign non-disclosure agreements, for example. By contrast, the OECD has publicly affirmed its commitment to ‘openness and transparency’ as ‘pillars for democracy, trust and progress’ (Gurria, 2016); a commitment that the NEA honours by making its workshop presentations available for download on its website. Should a member of the public be curious about how the *Workshop on Post-Accident Food Safety Science* reached its conclusions, they could access all 34 PowerPoint presentations and read all 623 slides, in either English or Japanese.^11^ The workshop sessions were also streamed via YouTube, albeit exclusively in English. Language skills permitting, any member of the public with internet access can watch all 19 hours, 17 minutes, and nine seconds of the workshop’s presentations, questions, and discussion. Where then—we might ask—is the ‘backstage’?

One can offer a partial answer by recognising that sheer volume can occlude public understanding. The hours of deliberation, hefty reports, and vast quantities of data made available by policy bodies are made more difficult to digest by the virtue of their super-abundance. And even if a committed citizen scientist or activist were to watch the NEA’s proceeding in their entirety, they would soon notice that the workshop is itself the product of unseen work. The staging of this three-day workshop rests on 18 months of planning, described by the organizers in only the most general terms. By cross-referencing statements made at the event with news items posted on the NEA website, it can be established that the workshop was first proposed by Fukuyama to NEA Director-General, William D. Magwood IV on 15 May 2015, during a visit to the NEA headquarters in Paris (Fukuyama, 2016; Inoue, 2016; OECD, 2015). A year later, Fukuyama’s successor, Shinji Inoue, made a follow-up visit, and was informed that the workshop was to be placed under the supervision of the NEA’s newly-appointed Deputy Director, Daniel Iracane (Inoue, 2016; OECD-NEA, 2016b). Beyond this, few details of the planning process can be ascertained. Ted Lazo’s role in organizing the logistics of the workshop, in his capacity as Scientific Secretary, goes largely unmentioned. Also absent are: any indication of how the scope of the workshop was agreed (i.e. a focus on reputational damage); the basis on which participants were chosen; and details of the guidance (if any) given to participants in preparing their presentations. Prosaic though these considerations may seem, their influence on the workshop’s capacity to stabilize a consensus are clear. In determining what is to be discussed by whom, the organizers are able to avoid contentious topics or phrases (e.g. radiophobia) and exclude ‘radical’ or ‘dissident’ voices—such as those of the anti-nuclear movement—who might disrupt proceedings. In so doing, the organizers exercise what Bachrach and Baratz (1962, 1963, 1970) termed ‘the second face of power’; limiting the scope of public discussion to a ‘consideration of only those issues [and opinions] which are comparatively innocuous’ to the convener (Bachrach & Baratz, 1970, p. 7).

One can also locate the ‘backstage’ within the workshop itself, drawing attention to what Crang (1994) terms the event’s ‘geographies of display’. Workshops are straightforwardly theatrical events. The audience, live and digital, is encouraged to direct their attention toward a lectern, from which participants give their presentations, and a projection of their PowerPoint slides. Audience members stand to ask a question—the camera swivelling toward them as a microphone is rushed to them by a member of the Cabinet Office. The audience politely ignores any ‘off-stage’ whispers and the clacking keys of participants discussing the proceedings via mail. Then, of course, there are the intermissions. Should a member of the general public choose to spend nine hours watching the first day of workshop proceedings, they would spend more than a fifth of their time staring at a holding slide, depicting nothing more than a crate of fish and the workshop details.^12^ This time is allocated to breaks—offering an opportunity for the participants to eat, drink, visit the lavatory, smoke, and converse more informally. No house curtain falls across the stage, but both camera and microphones are switched off, and the live audience dissipates. Following the broadcasting convention that ‘all places where the camera is not focused … or all places out of range of ‘live’ microphones’ are off-stage, the entire venue becomes a ‘back region’ (Goffman, 1959/1990).

To an observer, this shift between the ‘on stage’ and ‘off stage’ is immediate and obvious. The language is coarser, the statements more direct. One will hear claims dismissed with a colour and vigour unimaginable in the polite formality of a floor discussion: ‘It’s bullshit! Just bullshit!’^13^ In Goffman’s (1959/1990) terms, this ‘backstage’ region has different ‘requirements of decorum’ to the ‘front’ region, allowing participants to adopt a different ‘manner’ (pp. 110–111). Rather than interpreting this change of register as indicative of participants’ ‘authentic sel[ves]’ being revealed, as Goffman does (1959/1990, p. 115), I suggest that it marks a transition between performances. In the absence of a collective workshop-audience, myriad new dramaturgical teams are formed, performing as (and to) friends and colleagues. I found my own conversations with chairs and rapporteurs frequently interrupted by other participants, coming to inform my interlocutors of the resolution of some tension, scarcely mentioned in the formal floor discussions: ‘there was some confusion over [a given topic], but I think we managed to clear that up and achieve consensus’. Guided by an unspoken sense of professional courtesy, actors routinely backstaged their disagreements, allowing for the smooth consensus on food safety science to be performed frontstage. It is in these spaces that I first became aware of the disagreements between those who feel it appropriate to call the public radiophobic and those who do not.

## An improvised workshop

As we have seen, the metaphor of theatre helps us to identify a set of performative practices through which the OECD-NEA routinely performs its credibility. Focusing on the regimented elements of an organizational script can, however, lead the more contingent elements of its performance to be overlooked. In *Science on Stage*, for example, credibility contests unfold in a linear fashion. An expert body stages a claim to authority (Hilgartner, 2000, pp. 42-85), which comes under attack (pp. 86-112), prompting the organisation to mount a defence (pp. 113-145). If this team of actors is able to maintain dramaturgical cooperation, and continue speaking with a single voice, they succeed in repelling the attack. If they connot, they fail. Consequently, the central stake in the epistemic contest is whether the committee succeeds in sticking to the script.

Readers may already suspect that this account underplays the ‘performed resourcefulness’ of the NEA (Jeffrey, 2013). Certainly, one would expect all OECD workshops to employ ‘persuasive rhetoric’ of academic authority (Hilgartner, 2000, pp. 5-48)—but the choice of reputational damage over radiophobia is contextual. This choice anticipates the dramaturgical divisions that the (victim-blaming) psychological frame could create and nimbly avoids them. It is by choosing an economic register that the OECD can extend its vouching networks and speak with a single voice, while any mention of radiophobia is backstaged. So, the smooth staging of the workshop depends on actors’ ability to read their political and institutional situation and make rhetorical choices that permit dramaturgical cooperation.

The idiom of improvisation invites us to consider the emotional labour that the workshop’s organizers perform, not only to avoid pitfalls in staging, but to craft a persona that inspires trust. The distinction between staging and improvisation is not sharp, but elaborating their different emphases is instructive. In the previous section, I stressed how the economic frame facilitates co-operation between the actors; here, I focus on the specific roles they co-operate to perform. This moves the focus from the smoothness with which the script is staged, to the ingenuity with which it is adapted and embellished. Successful staging demands that a reading of the political context be stabilized between colleagues; effective improvisation requires that actors *read* the ‘emotional context’ of their audience, and embody roles that foster credibility, or pre-empt critique. My research informants may have accepted theatre as a *metaphor* for analysing practices of staging, but *self-consciously performed* the gestures I term improvisations. These acts were not a normal part of their professional personas, but roles and gestures adopted with specific (sceptical) audiences in mind.

The Japanese Cabinet Office’s decision to commission an international workshop, for example, can be read as a response to the hostility directed towards the Japanese state and the widely held conviction that the government safety standards served the interests of the ‘nuclear village’. Acknowledging the perceived conflict of interest that the state has in declaring Fukushima produce safe, the Cabinet Office opted to commission an external assessment. As Lazo notes, a credible expert is often imagined as ‘somebody from somewhere else’.^14^

… it often helps if they’re not invested in the situation that you’re having them talk about … in the workshops I’ve done here at the NEA since arriving in ’93, foreign experts tend to have more credibility than local experts, even though they might be saying the same thing. So, in that context … the fact that it was international was essential to being anything that anyone would possibly believe.

In a similar vein, Itoh emphasized how the workshop has brought together scientists from the ‘IAEA and United Nations Food and Agriculture Organization (UN-FAO) and other world, top-level scientists, from Japan and abroad’ to offer a ‘robust evaluation’ from their ‘*neutral* perspectives’ ([emphasis added] Itoh, 2016). Similarly, Inoue stressed that the workshop saw ‘top-level scientists in the world’ make their assessments of Japanese food safety standards ‘in good faith and based on science’ (Inoue, 2016). These claims of the international expert’s ability to take a ‘purely scientific approach’ to a complex political problem (Itoh, 2016), appear to be rooted in what Bourdieu (1975, p. 25) called the ‘naive philosophy of objectivity’: a frame of reference which inspires appeals to ‘international experts’—as if their position as foreign observers were sufficient to shield them from preconceptions and partisanship.

The value of a ‘view from somewhere else’ is made clear by the care with which the workshop stages a claim to being ‘international’ from predominantly Japanese human and financial resources. For although the workshop was convened by the NEA, it was commissioned by a Japanese Vice-Minister and hosted by the Japanese Cabinet Office on Japanese soil. Moreover, its selection of experts is predominantly Japanese. Not only were a majority of the 27 invited-speakers Japanese nationals, but half of the substantive sessions sported all-Japanese panels.^15^ To overcome this performative challenge, the organizers ensured that the most visible roles were filled by international experts. All Session Chairs, and most Vice-Chairs, are vouched for by an international organization: either the ICRP (8),^16^ NEA (1), or UN-FAO (1).^17^ The Chairs and rapporteurs^18^ are also ‘international experts’ in a second sense. Bearing names like Anne, Daniel and Rob, they hail from France, Germany, Netherlands, UK, and US. In short, they hail from a variety of nations, *except* Japan.^19^ This exclusion lends credence to the notion that it is the quality of providing a ‘view from somewhere else’ that is valued. The cast of the workshop remains largely Japanese, but the most visible roles (Chair and rapporteur) are filled by those who are in two different ways, ‘international’ (Table 3).

**Table 3.** National affiliations of presenters.

National affiliation	Session 1	Session 2	Session 3	Session 4	Total
Japan	6	7	0	4	17
UK	0	0	2	1	3
International	0	0	2	0	2
Belarus	0	0	0	1	1
Belgium	0	0	1	0	1
Germany	0	0	1	0	1
Netherlands	0	0	1	0	1
Norway	0	0	0	1	1

National affiliations as listed on the Workshop Programme (OECD-NEA, 2016a). Where no nation is given (i.e. for OECD-NEA or IAEA representatives), the individual has been counted as ‘international’.

Prior to the Fukushima Daiichi disaster, international agencies had enjoyed relatively limited authority in Japanese nuclear debates. Indeed, the insularity of Japan’s policy processes is commonly cited as the root cause of the disaster (NAIIC, 2012). The IAEA, for example, warned Japan of fundamental issues in its nuclear safety on multiple occasions, stressing the need to learn the lessons of Three Mile Island and Chernobyl (Funabashi & Kushner, 2015). Yet Japan proved sceptical of ‘anything not invented here’ and the council of the UN’s ‘nuclear watchdog’ was dismissed without widespread controversy (NAIIC, 2012, p. 7). Both critics and representatives of the state have often pointed to this failure to meet international standards as proof of regulatory capture in pre-Fukushima Japan. Famously, the former head of Japan’s Nuclear Safety Commission (NSC), Madarame Haruki, testified that regulatory capture had reduced the commission to thwarting international standards: ‘Though global safety standards kept improving, we wasted our time coming up with excuses for why Japan didn’t need to bother meeting them’ (in Tabuchi, 2012). The Cabinet Office’s decision to draw on an OECD assessment to close domestic debates about food safety science can therefore be seen as a departure from pre-2011 norms, reflecting both a recognition of the public’s hostility to the Japanese state and the rhetorical power of international standards. In (unfavourably) comparing Japanese regulations to international norms, critics were able to call the Japanese state’s credibility into question. Yet such attacks implicitly reinscribe these international norms as ‘gold standards’—allowing the state to counterattack by meeting (or exceeding) the very standards that their critics appealed to and commissioning international reviews of their food safety practices.

### International audiences

In addition to assuaging domestic fears, the workshop sought to inspire confidence in foreign audiences, whose impact on reputational damage is two-fold. First, foreign states and consumers directly influence the price of Fukushima produce. (Hong Kong and Taiwan continue to embargo Fukushima peaches, for example. Prior to 2011, they were the principal importers of this product, which is among Fukushima’s main agricultural exports.) Second, international audiences’ willingness to accept the safety of Fukushima produce has an indirect impact on domestic confidence. As one citizen scientist put it to me, ‘If food from Fukushima is so safe, why do so many nations continue to ban imports?’ Embargoes on food from Fukushima receive considerable attention in Japanese media outlets, ensuring that domestic audiences are keenly aware of foreign perceptions of Japanese food safety standards. And as the state’s case for the safety of Fukushima’s produce hinges on meeting (and exceeding) international requirements, foreign states’ (un)willingness to import food from Fukushima is imbricated with domestic perceptions.

Fukuyama (2016) makes clear his belief that the workshop had to be convened by an international organization to be credible oversees:

There are certain issues, domestically, here in Japan. There are academic societies in Japan. And there are those technical issues. But if we have some kind of discussion here in Japan, we may not be appreciated in the international arena. So, this OECD-NEA—as an international organization, we wanted to draw on your support. This international workshop is organized by the OECD-NEA as a result of such requests.

Fukuyama’s comments do not imply a hierarchy of competence between Japanese and international experts. To the contrary, he defends the technical sufficiency of Japan’s domestic expertise. Nonetheless, the Vice-Minister sees *strategic* value in commissioning an ‘international’ assessment. The decision to call upon the resources of the OECD can thus be seen as an expression of political ‘know-where’ (Agnew, 2007): the understanding that ‘where knowledge is produced and circulates is integral to its effects’ (Kuus, 2011b, p. 276; also Kuus, 2011a, 2015). It is ultimately because Fukuyama and his successors believe that an assessment bearing the stamp ‘Made by the OECD’ would circulate within the international community more readily than one anointed ‘Made in Japan’ that the workshop was organized.

At the time of the workshop, the stamp ‘Made by the OECD’ offered particular value in engaging two trading blocs—the Trans-Pacific Partnership (TPP) and EU—with whom Japan was negotiating and whose membership significantly overlaps with the OECD. On 4 February 2016, Japan joined 11 other states in the TPP. A majority of the partners, who collectively represented 40% of the global economy, are also OECD members.^20^ The credibility of Japan’s assurances was already accepted by eight of the partner nations, who had abandoned all restrictions on Japanese produce during the five-year negotiation of the treaty. However, the US was among the three exceptions. (It is therefore notable that the Workshop Chair was American.) The safety of food from Japan was less well accepted by the EU, which maintains particularly close ties with the OECD. All but five of the EU’s 27 member states are also OECD members and the EU maintains a permanent delegation at the OECD, so that it can ‘draw on the OECD’s unique reservoir of expertise’ (OECD, n.d.). The Japanese state therefore had good reason to believe that the credibility of an OECD assessment of food safety standards would be readily accepted within the EU. A close reading of workshop itinerary reveals that all scheduled participants were either from Japan, EU, US, or an international organization, with just one exception: a participant from Belarus, the nation most heavily affected by Chernobyl. Shortly after the workshop, the EU lifted its ban on rice produced in Fukushima (in 2016) and further eased its import restrictions in 2019. The three remaining states in the (unenforced) TPP have also either relaxed (US, Singapore) or abandoned (Brunei) their restrictions on Fukushima produce.

In thinking about how the credibility of the workshop was established with international audiences more broadly, one can productively take the notion of an ‘international community’ of radiation protection scientists literally. As in many specialist fields, the number of professionals working in radiological protection is relatively small. Consequently, attendance at conferences and workshops, as well as membership of committees, tends to overlap—creating close-knit networks of friendships and collegial relations. These connections cut across institutions and nations, as well as disciplines: this community of practice being composed of individuals trained in disciplines as diverse as physics, genetics, and ecology, as well as economics. The consequence is a field that is highly familiar and communal—something its members readily recognize. (At the invitation of a participant at the OECD-NEA workshop, I attended a NERIS workshop for the first time in 2017 and chose to present an analysis of the ‘nuclear village’ discourse [see Takahashi, 2017]. A lively Q&A was kicked-off by a question prefaced with the (joking) assertion that I was, in fact, addressing my analysis to ‘the international nuclear village’.) As Shapin (1995) notes, credibility often flows along such channels of familiarity. In such settings, ‘taking each other’s claims at face value is *normal*, and it is distrust, skepticism, and the demand for explicit warrants for belief that need to be justified and accounted for’ (Shapin, 1995, pp. 269-270, original emphasis). In keeping with Shapin’s insight, I observed that the case for a specific report or analysis’ credibility was frequently made with reference to the involvement of an acquaintance. Indeed, colleagues from Japanese ministries and agencies were often cited as a reason to treat specific assessments as credible even when some interlocutors voiced scepticism about the reliability of the Japanese state. The organization of an international workshop both maintains these networks and mobilizes them—the credibility of the workshop’s conclusions flowing through the personal networks of the participants into other international organizations and their respective states.

### The authority of witnessing

If objectivity is interpreted as synonymous with distance, why hold the conference in Fukushima? Why embed the production of knowledge in the local context, instead of holding it at the headquarters of the OECD-NEA in Paris? The graphs and tables that form the content of the presentations are, after all, ‘immutable mobiles’ that would read no differently in Paris than in Fukushima (Latour, 1987). And to locate the workshop in Fukushima, with the Japanese Cabinet Office acting as hosts, surely risks calling into question the very sense of distance that the organizers sought to cultivate in commissioning an international workshop. Though cost and convenience are certainly plausible factors, the workshop’s organizers noted that the choice of venue also had two performative functions.

The first was to stage the process of science communication in microcosm. ‘From the start, that [staging the workshop in Fukushima] was my suggestion’, Lazo recalls. ‘The idea was that if an international workshop was going to be of any value in providing information for people, it ought to be where the most people were concerned.’ To this end a two-hour public session was scheduled for the final day. Fewer than ten locals chose to attend. The 369 m² hall seats 360. Whether this was due to how the workshop was advertised or its timing (13.30 to 15.00 on a Thursday) is unclear, but the turnout was a source of disappointment for many participants. The session was always symbolic: an opportunity to perform the role of experts willing to engage directly with the public. But the public’s apparent disinterest derailed this effort to stage the workshop’s educational function.

As Vice-Governor Hata noted, conducting the workshop in Fukushima offered a second benefit in that it allowed participants ‘to see the situation [in Fukushima] first hand, particularly in regard to foodstuffs’ (Hata, 2016). On 7 November, a day prior to the workshop, attendees were offered a guided tour of four sites involved in the screening and distribution of Fukushima produce: Onahama Fish Market; Fukushima Agricultural Technology Centre; JA Fukushima Mirai, Sugitanouchi Warehouse; and Date Fruits Agricultural Cooperative Association. In visiting the stricken prefecture and seeing these facilities, participants took on the mantle of ‘witnesses’ to the effects of the disaster and ‘reputational damage’ upon the prefecture, able to make claims to knowledge derived from ‘a confrontation with the real, the “true”: the reality of the site’ (Wieviorka, 2006, p. 136). ‘I have seen people working so hard, so seriously’, Itoh announced on the first day of the workshop. ‘As a result of such low-profile and steady efforts, I am convinced that … safe products that have cleared the most stringent standards in the world are being distributed [from Fukushima]’ (Itoh, 2016). Delivered in sombre tones, the moral force of this personal reportage rests on the actors’ ability to perform their empathy (Hochschild, 1983); embodying the role of a politician or expert moved by the plight of the honest, hardworking people of Fukushima to organize the workshop. ‘[T]here is still—particularly in overseas countries—reputation[al] damage on food that is deeply rooted—that is *tormenting* [the] people of Fukushima’ (Itoh, 2016, emphasis added). In foregrounding the suffering which they have borne witness to, the organizers ask their audience to show the same compassion for the victims of reputational damage that they are performing and anticipate any suspicion of their motives. They enter the narrative of the workshop, not as self-interested members of the nuclear village, seeking to downplay the consequences of the nuclear disaster, but as witnesses to the human costs of consumer prejudice and allies to its victims.

Though the mantle of witness can be claimed by those observers who make a pilgrimage to Fukushima, Givoni (2013) reminds us that it is those who have endured the consequences of disaster that enjoy ‘unparalleled authority as a source of moral and political truth’ (p. 123). It is in this capacity that Minister Itoh invited seven students from *Soma Agricultural High School* to address the workshop. While the organizers spoke of what they had seen, the students spoke of that which they had lived, offering testimony, rather than analysis or reportage. Over the course of 20 minutes, the students described how they had been forced from their school in Soso, Minamisoma, relocating to a satellite campus in neighbouring Soma City. There, they spent two years waiting for the evacuation order to be lifted, returning to their school in 2013, just in time for its 110th anniversary. Since then, they told the assembled experts, they have devoted themselves to activities aimed at ‘sharing their energy’ with the community. Clubs dedicated to ritual forms of dance (including, Shinto ‘god performance’ (*kagura*) and ‘rice planting’ (*taue*) dances), and music (such as conch-shell music (*jingai*)) keep local traditions alive, to be enjoyed at an annual festival in October. Meanwhile, the 68-year-old Agriculture Club seeks to ‘cultivate loyalty and love’ for a farming region faced with reputational damage, by engaging in both practical research efforts and symbolic projects, such as making the fourth largest piece of ‘seed art’ in a Guinness World Record attempt: winning the 2014 Fan Choice award in the process. Unlike other participants, the seven presented together, taking it in turns to read paragraphs from a prepared statement, like members of a theatrical chorus. Acting in unison, they spoke *as* the Agricultural Club. The club, in turn, strives to ‘embody the hopes of the region’, they declared.^21^

This witness testimony became central to the workshop’s public persona despite the brevity of the students’ participation. They arrived for 14:00 on 8 November, delivered their speech, and left. No questions were directed toward them, and they had no opportunity to engage in the floor discussion. Yet the choreography of the workshop continually stressed their presence. They would be mentioned repeatedly in the opening remarks, conclusion, and press session and featured prominently in the local media’s reportage—a photo of the students being the only image that accompanied the Fukushima Minpo’s (2016) coverage of the event. Even the seating plan reflected their symbolic centrality, chairs having been reserved for the students in the middle of the hall, in rows six and seven of nine. (No other participants had seats allocated to them, save for those involved in chairing, who were seated at the front of the hall.) Subsequently, the students would become the focal point in how the workshop is remembered. On 3 May 2018, Minister Itoh visited the NEA’s headquarters to discuss the possibility of a follow-up to the workshop and to express his gratitude for the NEA’s work in Fukushima. ‘In particular,’ the NEA tells us, ‘the Minister expressed his thanks for the NEA’s support of the Soma Agricultural High School Students who took part in the workshop’ (OECD-NEA, 2018). These children’s testimony reminds us of the dramaturgical advantages of reputational damage over radiophobia. In separating the audience from the mantle of victimhood, it allows for emotive appeals from those directly affected (*tojisha*), whose presence renders a somewhat abstract economic problem ‘concrete enough to be apprehended’ (Hausner, in Wieviorka, 2006, p. 69).

### The experts’ bodies as evidence

The notion that ‘actions speak louder than words’ is an enduring article of folk wisdom and a common theme in popular works on authority and power, which urge readers to ‘win through your actions, never through argument’ (Greene, 1998). In deference to this principle, the experts expressed their confidence in Fukushima’s food safety standards, not just in their presentations, but in acts of gastronomy. This performance instrumentalized a prosaic ritual—the workshop dinner—by serving food and drink from Fukushima prefecture. Among the delicacies consumed were raw cuts of flounder (*hirame sashimi*), soba noodles and local wines (Fukushima Minpo, 2016). Unlike Sonoda, the NEA did not invite journalists to photograph them eating and drinking. However, they made numerous public references to their eating arrangements, ensuring that details of their meals reached local newspapers. In delivering his opening remarks, Daniel Iracane linked his own willingness to eat food ‘made in Fukushima’ to an awareness of the Japan’s management efforts:

Much research, practical implementation and monitoring has been done by the Japanese government, by food distributors, by farmers—to assure that any food produced from the Fukushima prefecture that reached the Japanese domestic market, or the international markets, or is locally consumed by those who grow it, meets rigorous governmental safety standards. *I am told that we will be eating Fukushima produced food today at this conference, and I am very happy to taste such high-quality delicacies* (Iracane, 2016, emphasis added).

Mike Boyd of the US EPA echoed this sentiment in announcing that those who made site visits on 6 November had felt sufficiently confident to eat Fukushima produce:

Many of us had the opportunity yesterday to taste the delicious food products from Fukushima prefecture, and we applaud the tremendous efforts that have been made to assure the world that these highly esteemed food products are safe and available (Boyd, 2016).

In eating Fukushima produce, the experts offered their own bodies as proof, not of the safety of the food *per se*, but of their sincerity in claiming to believe that it is so. The experts put their ‘skin in the game’ by performing the role of (literal) consumers to align their interests with those of their audience, not unlike the manager who buys stocks in her own company so as to align her interests with those of her shareholders (Taleb, 2017). ‘Science is supposed to be cold, straight, and detached’ (Latour, 2003, p. 208). Yet here, the credibility of expertise is rooted in the moral rectitude of the expert (Jasanoff, 1997, 2005). The gesture asks the audience to trust that food from Fukushima is safe on the grounds that these advisers are honest and ‘reliable’, rather than ‘(principally)’ appealing to the ‘rationality of their views’ (Jasanoff, 1997, p. 221). By demonstrating a willingness to tie their own health to the accuracy of their pronouncements, the experts tacitly make a case for their character and the ‘good faith’ nature of their assessments.

This gastronomic gesture is not new but remains a risky strategy. Jasanoff (1997, 2005) reminds us of the case of British Member of Parliament (MP), John Gummer. As concern that BSE—bovine spongiform encephalopathy or ‘mad cow disease’—had jumped the species barrier rose, Gummer attempted to assure the public that ‘British beef was safe to eat’. To do so, he fed a hamburger to his four-year-old daughter Cordelia, before the cameras of the assembled press. Far from inspiring confidence, the episode made Gummer an object of derision. The British press deemed him hungry for publicity and baulked at the cynicism of using his (unwitting) daughter as a political prop. Matters were not helped by Cordelia’s lack of enthusiasm. (The burger was too hot.) The episode underscores how easily dramaturgical gestures can backfire. And it was with reference to the memory of Gummer that the Anglophone press^22^ would report on Sonoda’s theatrics. Like poor Cordelia, they alleged, Sonoda had not mastered his role. ‘He confidently knocked back the glass of water in scenes reminiscent of John Gummer’, *The Independent* acknowledged; yet his ‘hand appeared to be shaking slightly as he poured the liquid’ (McNeill, 2011). The headline, ‘Trembling minister puts his Fukushima nuclear water where his mouth is’ does not inspire confidence (McNeill, 2011). Domestic opinion similarly focused on his moment of (apparent) hesitation. ‘Chicken’ (*bibiri*), one prominent comment reads on the video-streaming service, *Niko Niko Douga*. A satirical comic (*fūshiga*) by the illustrator Takeshi Kishino imagines Sonoda being hazed; sweating bullets as he forces the liquid down, surrounded by journalists chanting ‘Drink!’.

Nonetheless, Japanese public figures continue to stage the same piece of political theatre. Japan has now had four general elections and five Prime Ministers since the disaster, but the ritual of publicly eating Fukushima produce remains constant. Whether it be Cabinet Secretary Yukio Edano of the Democratic Party of Japan (DPJ) eating strawberries in Iwaki in 2011 or Prime Minister Shinzo Abe of the Liberal Democratic Party (LDP) eating Fukushima peaches in 2015, this appeal to common-sense continues. Abe also claimed to consume rice, milk, and yoghurt from Fukushima every day in the Prime Minister’s residence (Sankei Shimbun, 2016). The lesson that these public figures appear to have taken from Sonoda is that one should not wait to be challenged. Sonoda did not inspire a sense of security (*anshin*) because he was seen as buckling under public pressure. Yet any actor who declares Fukushima produce to be safe could face the same challenge. Sonoda agreed to drink the decontaminated water in response to repeated provocations from independent journalists; one of whom argued that Prime Minister Naoto Kan had established a ‘precedent’ (*zenrei*) earlier that year, when he ate white radish sprouts (*kaiware daikon*) from Fukushima (Asahi Shimbun, 2011). If the Prime Minister’s willingness to eat the sprouts could be accepted as proof that he believed it was safe, surely Sonoda’s unwillingness to drink the water could be taken as proof that he did not, the journalist reasoned. This common-sense line of attack resonated with the Japanese public. ‘If he [Sonoda] says its safe then making him drink it is reasonable (*datō*)’, one netizen encouraged (J-cast, 2011). In this context, proactively eating food from Fukushima appears to have emerged as a prophylactic maneuverer. Knowing that they are expected to embody the state’s confidence in food safety standards, actors pre-emptively emphasize their enjoyment of Fukushima produce, rather than risk being brow-beaten into submission. Treating the food’s safety as firmly established, Japanese politicians have tended to breeze past the technical language of becquerels per kilogram, to cheerily comment on the produce’s desirability. Abe said his Fukushima yoghurt had a ‘wonderfully rich (*taihen yutaka*) flavour’, for example (Sankei Shimbun, 2016). It is in keeping with this practice that the participants at the NEA workshop spoke of Fukushima produce as ‘delicious’ (Boyd, 2016) and ‘high-quality’ (Iracane, 2016). ‘We of the CRPPH look forward to enjoying these high-quality products in our own countries’, the workshop chair assured their audience (Boyd, 2016).

## Conclusion

In this article I have advanced the idiom of improvisation as a means to analyse how the Japanese state and OECD-NEA have performed their authority in conditions of low public trust. Their performance is both emotive and emotional: it aims to inspire a feeling of safety (*anshin*) toward food from Fukushima and relies on specific forms of emotional labour. The actors strive to achieve what the IAEA (1996) has termed the ‘management of emotions’ by interpreting their audiences’ ‘emotional context’ (p. 69) and fashioning an appropriate persona. In Japan, one common gesture has been to offer one’s own body as proof of one’s confidence in food safety standards. This personation of the state reflects the prominent role of common sense in Japan’s style of public reason and pre-empts the challenge that so vexed poor Sonoda: ‘if it’s safe, you consume it!’ Crucially, this effort to foil an attack on one’s credibility before it is made requires more than just empathy—it requires both the empathetic capacity to anticipate (effective) attacks and the dramaturgical ability to adapt one’s own performative practices. In contrast to the growing (idealist) literature on empathy in science communication, improvisation does not suggest that public trust is (necessarily) attained through the cultivation of (personal) virtue. As Greenblatt (1980) quips, ‘what others have called empathy, Shakespeare called Iago’ (p. 227). What is vital is not so much the virtue of the expert but their capacity to *perform* their virtue and/or nimbly deflect demands for further demonstration. Here, the distinction between the psychological discourse of ‘radiophobia’ (and the vernacular discourse of ‘radiation brain’) and the economic discourse of ‘reputational damage’ becomes clear. Where considerable scholarship on the Fukushima disaster has emphasized the overlap between these two incarnations of the deficit model, the lens of improvisation invites us to consider the dramaturgical advantages of the latter over the former. The shift from a discourse in which the audience are (irrational) *victims* to one in which the audience’s (irrational) fears *victimize* the people of Fukushima displaces the demand for empathy from the expert onto her audience. The implied moral obligation is no longer between expert and (psychologically distressed) audience but between both expert and audience toward the (economically stigmatized) people of Fukushima. By placing Fukushima farmers at the centre of the narrative in this way, the rhetoric of reputational damage also mitigates (populist) efforts to frame the debate as a struggle between the people and the ‘nuclear village’—allowing the experts to position themselves as allies of the most vulnerable, moved to act by the plight of reputational damage’s victims. Moreover, this discourse avoids the controversy associated with the term ‘radiophobia’, making the Japanese state more able to reach, and recruit the support of, international actors. In sum, the lens of improvisation allows us to see concrete differences in how the (psychological) discourse of radiophobia and (economic) discourse of reputational damage function, attuning us to the dramaturgical advantages of the latter over the former.

The discourse of reputational damage continues to be employed at the time of writing. In August 2023, Japan began to release ALPS-treated water from Fukushima Daiichi into the ocean, prompting China to embargo sea foods from Japan. Japanese Prime Minister Fumio Kishida responded by eating a sashimi lunch from Fukushima with three Cabinet Ministers (Francis & Inuma, 2023). The wave of domestic support for Fukushima fish (Yamaguchi, 2023), as well as the US military’s commitment to bulk-buy sea food from Fukushima (Hoskins, 2023), speaks to the continued rhetorical force of framing Fukushima’s food producers as victims of consumer irrationality (ARTE, 2024).

## Notes

1. UK media outlets who reported the story, for example, include: *The Guardian* (McCurry, 2011), *The Telegraph* (Demetriou, 2011), *The Independent* (McNeill, 2011), and *The Daily Mail* (Gardener, 2011)

2. ‘Government patronized scholar’ would be a more literal translation but ‘academic flunky’ has become standard in English language publications on the Fukushima disaster and better captures the pejorative intent of the term.

3. Calculated on the basis of data compiled by the EC et al. (1996).

4. The objectives of the workshop were to: (1) ‘present the state-of-the-art scientific aspects of post-accident food safety, including: radionuclide transfer to foodstuffs and dose modelling; agricultural management to reduce food contamination; and measurement techniques’ (2) ‘present the status of and the remaining challenges related to local, national and international management of post-accident food safety’; and (3) ‘discuss approaches for addressing remaining challenges’ (NEA, 2016).

5. On 17 March 2011, the Department of Food Safety (Ministry of Health, Labour and Welfare (MHLW)) adopted the ‘Indices relating to limits on food and drink ingestion’, published by the Nuclear Safety Commission (NSC) of Japan. This framework established thresholds for contamination in drinking water (200 bq/kg), milk and other dairy products (200 bq/kg), vegetables (500 bq/kg), grains (500 bq/kg), and ‘meat, fish, eggs, etc.’ (500 bq/kg).

6. Under the 2012 framework, thresholds for contamination are set at: 10 bq/kg in drinking water, 50 bq/kg in milk, and 100 bq/kg in ‘general foods’, such as meat, vegetables, and eggs.

7. A planned 15 hours 10 minutes of discussion was filmed. Given the simultaneous translation, I conservatively estimate the rate of speech at 100 words per minute.

8. *Fūhyo* is difficult to translate. I used the approximation ‘harmful rumour’ here as this was the translation used at the OECD NEA workshop. This translation has become common in both the academic and policy literatures. However, it should be noted that much of this literature cites Sekiya (2003, 2011) as offering the authoritative definition of reputational damage. Sekiya (2011) stresses that *fūhyohigai* is ‘not attributable to rumour generated by human communication but a product of media blitz’ (p. 93). The terminology thus draws a distinction between those living in the affected areas, who may spread rumours, and ‘those who are outside the disaster-striken area [and] are affected by … media reports [who] cause this fuhyohigai’.

9. 放射脳

10. 放射能

11. All but six of the presentations are available in Japanese.

12. The breakdown of time allocation on 8 November 2016 is as follows: 50 minutes of opening remarks and introductions (9%); six hours 25 minutes of presentations (71%); 80 minutes of floors discussion (15%) and two hours of breaks (22%).

13. Field Journal, 8 November 2016.

14. Lazo, Interviewed July 2017

15. This calculation distinguishes the 27 speakers who delivered substantive presentations, in Sessions One to Four, from the four rapporteurs who offered summaries in Session Five and are discussed on the following pages.

16. Six are members of an ICRP Task Group and four are members of ICRP Committee 4.

17. The exceptions are Hirofumi Tsudaka and Naoya Sekiya, both of whom hail from reputable universities (Fukushima University and Tokyo University, respectively).

18. Rapporteurs offered summaries of the four main sessions on the third day of the workshop in Session Five.

19. In contrast to the Chairs, every Vice-Chair is Japanese. This nationalized division of labour is strictly maintained, despite uneven numbers. Thus, two ‘foreign’ experts (namely, Iracane and Theelan) chaired Session Two together, while Michiaki Kai acted as Vice-Chair on two separate sessions (Sessions Three and Four).

20. Ultimately, the TPP was not ratified. US President Donald Trump would later withdraw the US from the agreement.

21. Itoh echoed this rhetorical move, emphasising that it was ‘indispensable to have the presence of high-school students’, as they were the ‘backbone of the food industry in Fukushima in the future’ (Itoh, 2016).

22. Comparisons of Sonoda to Gummer appeared in British press outlets, such as *The Independent* (McNeill, 2011), *The Guardian* (McCurry, 2011), The Times (2011), and the *Daily Mail* (Gardener, 2011), as well as in the *Irish Independent* (2011), the *Australian Broadcasting Corporation* (Willacy, 2011), and the *Sydney Morning Herald* (Demetriou, 2011)).
